# Assessing COVID-19 Vaccine Hesitancy, Confidence, and Public Engagement: A Global Social Listening Study

**DOI:** 10.2196/27632

**Published:** 2021-06-11

**Authors:** Zhiyuan Hou, Yixin Tong, Fanxing Du, Linyao Lu, Sihong Zhao, Kexin Yu, Simon J Piatek, Heidi J Larson, Leesa Lin

**Affiliations:** 1 School of Public Health Fudan University Shanghai China; 2 National Health Commission Key Laboratory of Health Technology Assessment Fudan University Shanghai China; 3 Global Health Institute Fudan University Shanghai China; 4 Department of Infectious Disease Epidemiology London School of Hygiene & Tropical Medicine London United Kingdom; 5 Laboratory of Data Discovery for Health Hong Kong Science Park Hong Kong SAR China

**Keywords:** COVID-19 vaccine, hesitancy, infoveillance, infodemiology, confidence, acceptance, engagement, social media, COVID-19

## Abstract

**Background:**

Monitoring public confidence and hesitancy is crucial for the COVID-19 vaccine rollout. Social media listening (infoveillance) can not only monitor public attitudes on COVID-19 vaccines but also assess the dissemination of and public engagement with these opinions.

**Objective:**

This study aims to assess global hesitancy, confidence, and public engagement toward COVID-19 vaccination.

**Methods:**

We collected posts mentioning the COVID-19 vaccine between June and July 2020 on Twitter from New York (United States), London (United Kingdom), Mumbai (India), and Sao Paulo (Brazil), and Sina Weibo posts from Beijing (China). In total, we manually coded 12,886 posts from the five global metropolises with high COVID-19 burdens, and after assessment, 7032 posts were included in the analysis. We manually double-coded these posts using a coding framework developed according to the World Health Organization’s Confidence, Complacency, and Convenience model of vaccine hesitancy, and conducted engagement analysis to investigate public communication about COVID-19 vaccines on social media.

**Results:**

Among social media users, 36.4% (571/1568) in New York, 51.3% (738/1440) in London, 67.3% (144/214) in Sao Paulo, 69.8% (726/1040) in Mumbai, and 76.8% (2128/2770) in Beijing indicated that they intended to accept a COVID-19 vaccination. With a high perceived risk of getting COVID-19, more tweeters in New York and London expressed a lack of confidence in vaccine safety, distrust in governments and experts, and widespread misinformation or rumors. Tweeters from Mumbai, Sao Paulo, and Beijing worried more about vaccine production and supply, whereas tweeters from New York and London had more concerns about vaccine distribution and inequity. Negative tweets expressing lack of vaccine confidence and misinformation or rumors had more followers and attracted more public engagement online.

**Conclusions:**

COVID-19 vaccine hesitancy is prevalent worldwide, and negative tweets attract higher engagement on social media. It is urgent to develop an effective vaccine campaign that boosts public confidence and addresses hesitancy for COVID-19 vaccine rollouts.

## Introduction

As of January 2021, the COVID-19 pandemic has led to more than 100 million cases and 2 million deaths worldwide [1]. Although personal prevention measures such as mask wearing and social distancing have been shown to be effective in curbing the spread of COVID-19 [2], vaccination is expected to be the key to the long-term prevention and control of the pandemic [3,4]. The COVID-19 pandemic has triggered intense global research and development (R&D) of vaccines against the disease. Several candidate vaccines advanced to Phase III clinical trials in mid-2020, including the Oxford/AstraZeneca, Sinopharm, Sinovac, BioNTech/Pfizer, and Moderna vaccines, and had finished clinical trials at the end of 2020. These vaccines have been approved for use by December 2020 in some countries such as the United Kingdom, the United States, and China [5]. To ensure universal vaccination coverage, governments must enhance public confidence, address the issue of vaccine hesitancy, and design community engagement strategies for COVID-19 vaccine rollouts.

Although immunization has proved successful in reducing the global burden of illness and death, a range of concerns have converged to affect public confidence in vaccines. When vaccine confidence breaks down, hesitancy can cause serious consequences such as delays, refusals, and disruptions to research and delivery programs, and sometimes leads to the resurgence of disease outbreaks [6]. Vaccine hesitancy has proliferated over the decades and was cited by the World Health Organization (WHO) as one of the top 10 global health threats in 2019 [7,8]. Vaccine hesitancy is complex, multifactorial, and influenced by a combination of emotional, cultural, social, spiritual, and political factors. It can vary across countries, vaccines, and time. Reports have indicated that hesitancy toward general vaccines is prevalent among caregivers of children worldwide, at rates such as 45.8% in France (2016) [7], 31.8% in the United States (2014) [9], 24.6% in Italy (2017) [10], and 23% in Brazil (2016) [11]. The prevalence of vaccine hesitancy among health care providers ranges from 2% to 16% across different countries [12]. The accelerated R&D process of the COVID-19 vaccine may further exacerbate public concern on its safety and effectiveness [13]. Similarly, the novelty of the disease, politicization of the vaccine, and distrust in experts and governments have increased uncertainty about COVID-19 vaccination [3]. Therefore, it is necessary to assess public confidence and acceptance toward the COVID-19 vaccine to prepare for vaccine introduction.

Social media has become a source of data for detecting outbreaks and understanding public attitudes and behaviors during public health emergencies [14-16]. Large amounts of real-time data posted on social media platforms can be used to quickly identify public attitudes on COVID-19 vaccines as a way to support health communication and health promotion messaging. A growing body of literature has used social media platforms such as Twitter and Facebook for public health research [15]. Compared with traditional surveys, social media listening can not only monitor public attitudes in a timely manner but also assess the dissemination of and public engagement with these opinions [17]. Individuals are increasingly using social media to communicate with each other, and public engagement can assess how various messages around COVID-19 vaccines spread on social media. Although several previous studies investigated public acceptance toward COVID-19 vaccines using questionnaire surveys, it is unknown how these opinions spread among the public.

By using social media listening data from the largest metropolises worldwide, this study aimed to assess global vaccine hesitancy and confidence toward COVID-19 vaccination and public engagement in communications about COVID-19 vaccines.

## Methods

### Data Collection

Twitter is one of the most popular social media platforms in the world; Sina Weibo, Twitter in China, is the most influential social media platform in China, with over 500 million users. Users can share information or opinions by tweets or posts on these platforms. Using the Meltwater platform [18], we collected posts mentioning COVID-19 vaccines on Twitter from New York (United States), London (United Kingdom), Mumbai (India), Sao Paulo (Brazil), and Sina Weibo posts from Beijing (China). The five metropolises were selected due to their high disease burden of COVID-19. The data covered the period from June 13 to July 31, 2020, when five COVID-19 vaccines started their Phase III clinical trials worldwide, including the Oxford/AstraZeneca, Sinopharm, Sinovac, BioNTech/Pfizer, and Moderna vaccines [19]. The keywords included “COVID vaccin*” OR “COVID-19 vaccin*” OR “COVID19 vaccin*” OR “coronavirus vaccin*” OR “vaccin* for coronavirus” OR “vaccin* for COVID.” Each post record comprised account name, contents, post time, the number of followers, and engagement data. Since our study aimed to assess public attitudes toward COVID-19 vaccination, we only included tweets or Weibo posts from individual accounts and excluded those from news and organizational accounts. Duplicate tweets, tweets with identical text but different tweet identifications, retweets, quotes without comments, and irrelevant tweets were removed [20]. This study was exempt from ethical review because it examined retrospective publicly available data.

### Content Analysis

We identified and classified posts describing personal opinions or discussion of COVID-19 vaccines from Twitter and Sina Weibo. A coding framework (Multimedia Appendix 1) was developed for content analysis according to the WHO’s Confidence, Complacency, and Convenience (“3 Cs”) model of vaccine hesitancy [21] and validated through manual annotation of the subset with 500 posts. All posts were double-coded independently, and a third coder resolved disagreements. Posts were initially classified as relevant or irrelevant to personal opinions toward COVID-19 vaccines, and relevant posts were further classified to the predefined categories in Multimedia Appendix 1. Predefined categories included attitudes toward COVID-19 vaccination (accept, neutral, doubt, or refuse), expectations of COVID-19 vaccine R&D and introduction (positive, neutral, or negative), confidence in COVID-19 vaccine importance (important or not), confidence in COVID-19 vaccine effectiveness (effective or not), confidence in COVID-19 vaccine safety (safe or not), trust in governments (trust or not), trust in experts (trust or not), misinformation or rumors about all vaccines, complacency (perceived risk of getting COVID-19: high or low), COVID-19 vaccine convenience (accessibility, distribution, or affordability), COVID-19 vaccine types (AstraZeneca, Moderna, Pfizer, or Chinese vaccines), and others. Each post could be classified as one category, multiple categories, or no category. We described simple counts and percentages of posts for each topic on COVID-19 vaccination.

### Social Media Engagement Analysis

We also conducted social media engagement analysis to investigate public communication and interaction about various topics relating to the COVID-19 vaccine online. In Twitter, the engagement metric measures all actions viewers have taken as a result of seeing tweets and engaging with tweets [22]. At the level of public engagement and interaction, Twitter engagement refers to retweets, follows, replies, favorites, and clicks on the tweets. It covers the three metrics of social media engagement: popularity based on the number of likes for tweets, commitment based on the number of comments for tweets, and virality based on the number of shares for tweets [22]. The mean number and SD of engagement with and followers of tweeters were presented for each topic of COVID-19 vaccines.

## Results

### Description of Analyzed Social Media Posts

**Figure 1:**
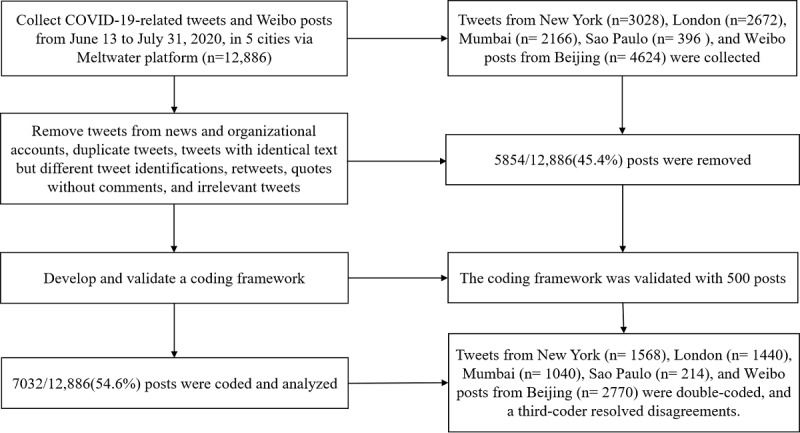
Flowchart of data process and analysis.

Figure 1 shows the process of data selection and analysis. In total, we collected 12,886 social media posts mentioning a COVID-19 vaccine. There were 3028 tweets on Twitter from New York, 2672 tweets from London, 2166 tweets from Mumbai, 396 tweets from Sao Paulo, and 4624 Sina Weibo posts from Beijing. After assessment, 7032 posts met the inclusion criteria and were included in our content analysis, including 1568 tweets from New York, 1440 tweets from London, 1040 tweets from Mumbai, 214 tweets from Sao Paulo, and 2770 Sina Weibo posts from Beijing.

### COVID-19 Vaccine Hesitancy and Confidence

**Figure 2:**
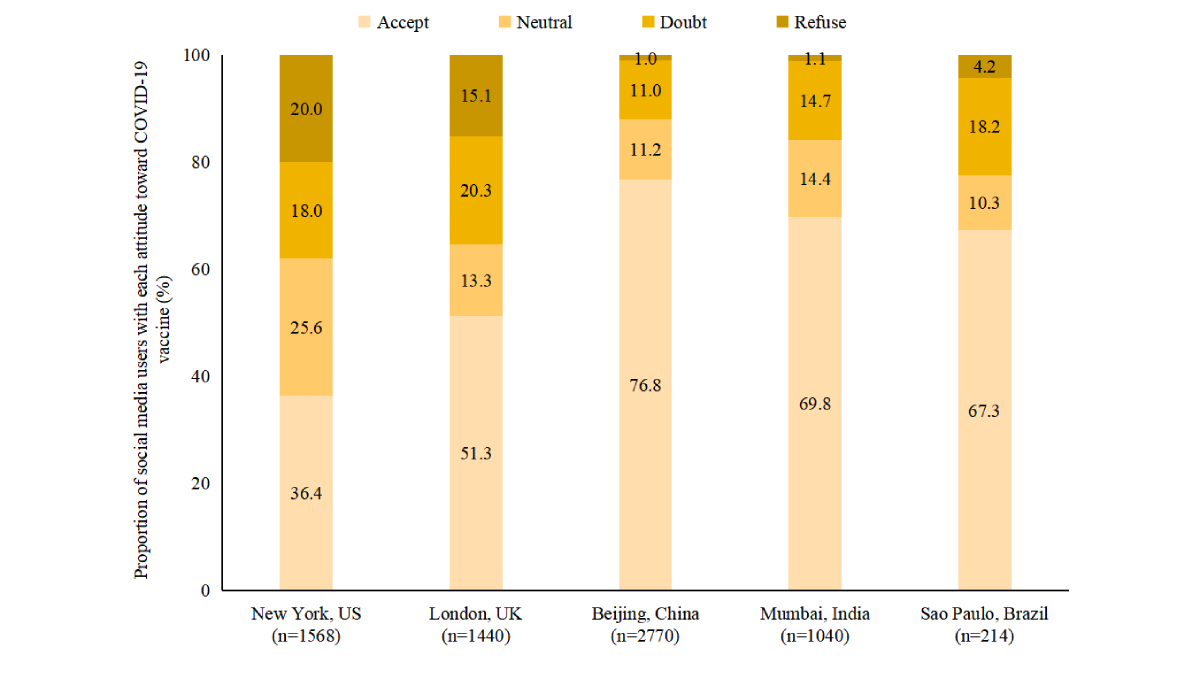
Social media users’ attitudes toward COVID-19 vaccination.

**Figure 3:**
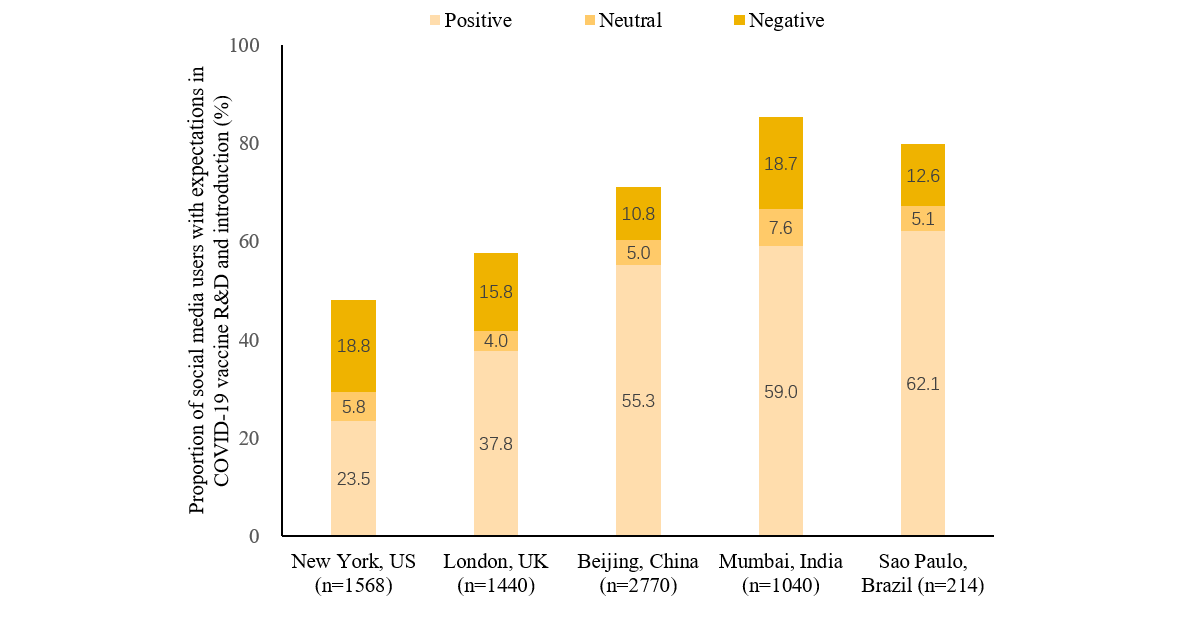
Expectations of COVID-19 vaccine R&D and introduction. R&D: research and development.

Figure 2 presents social media users’ attitudes toward COVID-19 vaccination. Among social media users, 36.4% (571/1568) in New York and 51.3% (738/1440) in London reported willingness to accept COVID-19 vaccines, much lower than observed in metropolises in lower- and middle-income countries (LMICs), such as 69.8% (726/1040) in Mumbai, 67.3% (144/214) in Sao Paulo, and 76.8% (2128/2770) in Beijing. Although about 10% to 20% of users doubted the safety or effectiveness of COVID-19 vaccines in each metropolis, 20% (313/1568) in New York and 15.1% (218/1440) in London expressed refusal, much higher than other metropolises (<5%), leading to prevalent vaccine hesitancy. About 50% to 80% of users discussed the R&D and introduction of the COVID-19 vaccine globally, with most showing positive expectations, and the remaining users did not mention the R&D and introduction of COVID-19 vaccines (Figure 3). The discussion levels on the R&D and introduction of the COVID-19 vaccine in New York and London were lower than those in Mumbai, Beijing, and Sao Paulo.

Table 1 conveys confidence, complacency, and convenience related to COVID-19 vaccination on social media. Although most tweets in each metropolis regarded COVID-19 vaccination as important and effective, proportions of tweets perceiving COVID-19 vaccines as unsafe in high-income countries (HICs; 133/1568, 8.5% in New York and 157/1440, 10.9% in London) were much higher than those in other metropolises (<4%). These HIC users expressed distrust in governments (>10%) and experts (around 5%). Furthermore, 11.8% (185/1568) and 11% (158/1440) of users in New York and London, respectively, mentioned misinformation, rumors, or antivaccine campaigns, but few users in LMIC metropolises (<7%) shared these concerns.

**Table 1:** Confidence, complacency, and convenience related to COVID-19 vaccines on social media in 2020.

Topics						New York, US			London, UK			Beijing, China			Mumbai, India			Sao Paulo, Brazil
**Total posts^a^, n**						3028			2672			4624			2166			396
	Irrelevant posts				1460			1232			1854			1126			182	
	Relevant posts				1568			1440			2770			1040			214	
**Topics among relevant posts^b^, n (%)**																		
	**Vaccine confidence**																	
		**Perceived importance of vaccines**			115 (7.3)			130 (9.0)			712 (25.7)			140 (13.5)			22 (10.3)	
			Important	96 (6.1)			115 (8.0)			651 (23.5)			132 (12.7)			22 (10.3)		
			Unimportant	19 (1.2)			16 (1.1)			61 (2.2)			8 (0.8)			0 (0.0)		
		**Perceived effectiveness of vaccines**			217 (13.8)			361 (25.1)			928 (33.5)			294 (28.3)			71 (33.2)	
			Effective	142 (9.1)			305 (21.2)			674 (24.3)			261 (25.1)			68 (31.8)		
			Ineffective	75 (4.8)			56 (3.9)			254 (9.2)			32 (3.1)			3 (1.4)		
		**Perceived safety of vaccines**			168 (10.7)			354 (24.6)			201 (7.3)			190 (18.3)			47 (22.0)	
			Safe	35 (2.2)			197 (13.7)			143 (5.2)			149 (14.3)			43 (20.1)		
			Unsafe	133 (8.5)			157 (10.9)			58 (2.1)			41 (3.9)			4 (1.9)		
		**Trust in governments**			294 (18.8)			177 (12.3)			148 (5.3)			72 (6.9)			8 (3.7)	
			Trust	14 (0.9)			19 (1.3)			138 (5.0)			16 (1.5)			1 (0.5)		
			Distrust	280 (17.9)			158 (11.0)			10 (0.4)			56 (5.4)			7 (3.3)		
		**Trust in experts**			90 (5.7)			111 (7.7)			—^c^			78 (7.5)			2 (0.9)	
			Trust	14 (0.9)			44 (3.1)			—			24 (2.3)			2 (0.9)		
			Distrust	76 (4.8)			67 (4.7)			—			54 (5.2)			0 (0.0)		
	**Information around vaccines**																	
		Misinformation or rumors			185 (11.8)			158 (11.0)			188 (6.8)			30 (2.9)			12 (5.6)	
	**Complacency**																	
		**Perceived risk of getting** **COVID-19**			89 (5.7)			52 (3.6)			742 (26.8)			45 (4.3)			4 (1.9)	
			High	80 (5.1)			45 (3.1)			688 (24.8)			41 (3.9)			4 (1.9)		
			Low	9 (0.6)			7 (0.5)			54 (1.9)			4 (0.4)			0 (0.0)		
	**Vaccine convenience**																	
		Vaccine accessibility			94 (6.0)			76 (5.3)			283 (10.2)			99 (9.5)			49 (22.9)	
		Vaccine distribution			309 (19.7)			261 (18.1)			325 (11.7)			63 (6.1)			26 (12.1)	
		Vaccine affordability			107 (6.8)			40 (2.8)			197 (7.1)			33 (3.2)			20 (9.3)	
	**Vaccine types**				259 (16.5)			470 (32.6)			941 (34.0)			270 (26.0)			122 (57.0)	
		AstraZeneca			102 (6.5)			388 (26.9)			82 (3.0)			218 (21.0)			63 (29.4)	
		Moderna			51 (3.3)			30 (2.1)			37 (1.3)			31 (3.0)			21 (9.8)	
		Pfizer			88 (5.6)			26 (1.8)			34 (1.2)			18 (1.7)			27 (12.6)	
		Chinese vaccines			25 (1.6)			26 (1.8)			831 (30.0)			19 (1.8)			13 (6.1)	
	**Others**				83 (5.3)			51 (3.5)			990 (35.7)			34 (3.3)			5 (2.3)	

^a^We assessed 50% random samples from New York and London due to the large sample size, and full samples in Beijing, Mumbai, and Sao Paulo.

^b^Topics are calculated among relevant posts.

^c^Trust in experts is not measured specifically for Sina Weibo posts from Beijing.

Regarding complacency, most social media users perceived the risk of getting COVID-19 to be high despite only a small number of them (around 5% except Beijing) directly mentioning the risk of getting COVID-19. For vaccination convenience, approximately 5% of HIC users mentioned vaccine accessibility, whereas more users in LMIC metropolises (10%-20%) worried about vaccine accessibility, including production and supply capacity for COVID-19 vaccines. Nearly 20% of HIC users worried about vaccine distribution, including priority vaccination groups, whereas less users in LMIC metropolises (around 10%) mentioned this subject. Few users (about 5%) considered vaccine affordability in mid-2020. There were differential concerns on vaccine accessibility worldwide.

The proportion of tweets mentioning specific vaccine types was 16.5% (259/1568) in New York, 57% (122/214) in Sao Paulo, and around 30% in the remaining three metropolises. Tweets in New York mainly discussed COVID-19 vaccines produced in the United States, and tweets in other metropolises discussed vaccines produced worldwide. In terms of specific COVID-19 vaccines that had started Phase III clinical trials, social media users in metropolises except for Beijing mostly mentioned the Oxford/AstraZeneca vaccine, followed by the BioNTech/Pfizer and Moderna vaccines; the Chinese vaccine was the least frequently mentioned. This indicated that public attention was consistent to the development progress of specific vaccines.

### Engagement Levels of COVID-19 Vaccine–Related Tweets

Table 2 presents the engagement metrics of COVID-19 vaccine–related tweets by topic. On average, tweeters who posted COVID-19 vaccine–related tweets had 3634 followers, and their followers ranged from 1637 (expressing COVID-19 vaccination is important) to 4778 followers (expressing COVID-19 vaccination is unsafe) and 7430 followers (expressing COVID-19 vaccination is ineffective). Overall, tweeters expressing negative attitudes toward COVID-19 vaccines, such as doubt or refusal to vaccinate, lack of confidence in vaccines (importance, effectiveness, and safety), and misinformation or rumors, had more followers than those expressing positive attitudes.

COVID-19 vaccine–related tweets attracted 6.1 engagement values on average. When comparing engagement levels on different topics regarding COVID-19 vaccines, we found that the most active topics were misinformation (engagement value 14.0), followed by confidence in vaccine safety (13.1), vaccine effectiveness (10.9), and governments (9.7), whereas the least active topics were vaccine convenience (2.6-5.8), confidence in vaccine importance (3.6) and experts (3.8), and complacency (5.4). For topics on vaccine hesitancy and expectations in vaccine R&D and introduction, tweets with clear attitudes attracted more engagement than neutral tweets, but there was little difference in public engagement between tweets with positive and negative attitudes. For each topic regarding vaccine confidence except distrust in governments, lack of confidence propagated more than having confidence, especially for vaccine safety (16.7 engagement for tweets expressing vaccine is unsafe vs 10.3 engagement for tweets expressing vaccine is safe); trust in governments attracted much more engagement than distrust (52.8 vs 5.4), representing the public’s common expectations of governments taking an active role in controlling the epidemic.

**Table 2:** Followers and engagements of COVID-19 vaccine–related tweets in 2020.

Topics				Followers, mean (SD)		Engagements, mean (SD)
**Vaccine hesitancy**						
	**Attitudes toward COVID-19 vaccination**			3633.6 (12,614.4)		6.1 (61.4)
		Accept	2448.8 (8614.6)		7.9 (93.3)	
		Neutral	4333.0 (16,250.7)		5.8 (28.5)	
		Doubt	4593.3 (18,245.7)		10.0 (93.4)	
		Refuse	2708.4 (12,504.3)		6.4 (35.4)	
	**Expectations of COVID-19 vaccine R&D^a^ and introduction**			3011.0 (11,759.4)		8.4 (94.4)
		Positive	2527.7 (9083.2)		8.8 (104.3)	
		Neutral	4449.8 (16,399.9)		5.9 (31.9)	
		Negative	3629.4 (14,833.2)		8.2 (84.0)	
**Vaccine confidence**						
	**Perceived importance of vaccines**			1763.6 (3727.9)		3.6 (10.7)
		Important	1636.5 (3167.2)		3.4 (9.9)	
		Unimportant	2843.1 (6811.7)		5.4 (16.5)	
	**Perceived effectiveness of vaccines**			3373.4 (13,960.7)		10.9 (115.5)
		Effective	2505.6 (8366.8)		10.6 (119.5)	
		Ineffective	7430.1 (27,615.6)		12.2 (94.7)	
	**Perceived safety of vaccines**			3274.3 (14,058.0)		13.1 (137.2)
		Safe	2086.2 (6191.7)		10.3 (137.4)	
		Unsafe	4778.0 (19,897.0)		16.7 (137.1)	
	**Trust in governments**			3865.2 (16,363.8)		9.7 (111.8)
		Trust	3055.6 (6573.5)		52.8 (360.4)	
		Distrust	3946.0 (17,036.6)		5.4 (28.7)	
	**Trust in experts**			1887.9 (4812.1)		3.8 (14.7)
		Trust	2029.1 (4076.9)		3.0 (6.5)	
		Distrust	1827.7 (5102.0)		4.1 (17.1)	
**Information around vaccines**						
	Misinformation or rumors			3127.7 (11,610.5)		14.0 (121.2)
**Vaccine complacency**						
	**Perceived risk of getting COVID-19**			3017.9 (8282.9)		5.4 (15.6)
		High	2909.7 (7848.9)		5.1 (15.5)	
		Low	3937.6 (11,554.1)		8.1 (16.5)	
**Vaccine convenience**						
	Vaccine accessibility			2351.5 (8915.4)		2.6 (7.8)
	Vaccine distribution			3531.5 (12,541.6)		5.8 (26.2)
	Vaccine affordability			2525.3 (4895.6)		3.1 (8.3)

^a^R&D: research and development.

## Discussion

### Principal Findings

This social listening study in large metropolitan areas in five countries examined hesitancy toward COVID-19 vaccination; perceptions of vaccine confidence, complacency, and convenience; and level of online public engagement. We found that COVID-19 vaccine hesitancy was prevalent worldwide, with the highest prevalence in New York and London, followed by Sao Paulo and Mumbai, and the lowest in Beijing. With high perceived risk of getting COVID-19, social media users in HICs, including the United States and the United Kingdom, expressed low acceptance of COVID-19 vaccines, serious concerns regarding vaccine safety, and distrust in governments and experts. There were different concerns about vaccination convenience and accessibility between HICs and LMICs. Negative tweets expressing lack of vaccine confidence and misinformation or rumors had more followers and attracted more online public engagement.

Overall, social media users expressed relatively high hesitancy toward COVID-19 vaccination across metropolises with high COVID-19 burdens. There were no metropolises where willingness to accept a COVID-19 vaccine exceeded 80%, and only between 36% to 50% of tweeters accepted the COVID-19 vaccine especially in New York and London. These social listening results were similar to global survey studies [13,23], which showed that COVID-19 vaccine acceptance was higher in China, India, and Brazil but lower in the United States and the United Kingdom. Over the past decade, vaccine refusal has accelerated worldwide, and antivaccine activities have been amplified through political activities and social media [24]. The prevalent hesitancy toward COVID-19 vaccines could potentially lead to low vaccination coverage, which will further delay global control of the pandemic and societal and economic recovery. Recent studies estimated a COVID-19 R_0_ of around 3.87 for Europe and around 3.45 for the United States [25,26], implying herd immunity thresholds of 74% and 71%, respectively. The current acceptance levels among tweeters in New York, London, Mumbai, and Sao Paulo is insufficient to reach this 71% to 74% threshold; one exception is Beijing with an acceptance level at 76.8%. As governments are preparing to introduce COVID-19 vaccines and initiate postpandemic recovery, the need to develop an effective vaccine campaign for the rollout that boosts public confidence and addresses hesitancy is urgent.

A previous study showed that confidence in vaccines and governments was strongly associated with vaccine acceptance and uptake [13]. However, our findings showed lack of confidence in vaccine safety, distrust in governments and experts, and widespread misinformation or rumors, especially in HICs. Public confidence in the safety and effectiveness of COVID-19 vaccines was far lower than the confidence level in general vaccines. In the 2018 Global Monitor Survey, 79% and 84% of the public agreed that vaccines were safe and effective globally [27]. During COVID-19, the development of a vaccine for a new pathogen has been pushed much faster than ever before, and new bioscience technologies (eg, mRNA vaccine) are being used in humans for the first time [5,28]. In light of the accelerated R&D process, any negative news related to vaccine failure may weaken public confidence in its safety and effectiveness [13,29]. Therefore, effective communication campaigns should be designed to explain the safety of COVID-19 vaccines to the public and clarify misinformation or rumors, especially in HICs. Communication campaigns should be supported by the scientific community to address public concerns in COVID-19 vaccines. Through creating a space for a collaborative dialogue between the scientific community and the public, these campaigns would aim to not only provide the public with the latest information but also build public confidence in vaccine programs.

Social media users in HICs and LMICs expressed different concerns about vaccination convenience and accessibility. HIC users had less concerns about production and supply capacity of COVID-19 vaccines but more concerns on vaccine distribution and inequity. In contrast, LMIC users worried more about vaccine production and supply instead of vaccine distribution. Although HICs can ensure the supply of COVID-19 vaccines, systemic racial and economic disparities have existed for a long time in many fields, including health care and vaccination. The COVID-19 pandemic has disproportionately affected low-income groups and communities of color [24]. Disparities in access to COVID-19 vaccines possibly still occur and may be a point of concern that needs to be addressed in HICs. There is also much concern regarding access to COVID-19 vaccines and rollout gaps in LMICs compared with HICs. Many HICs have sought to gain priority access to COVID-19 vaccines by striking advance purchase agreements with vaccine manufacturers, instead of through WHO’s global allocation mechanism, leaving few vaccine doses for LMICs [23,30]. Therefore, vaccine campaigns should be tailored to each context to address local concerns: LMICs should take efforts to address vaccine supply issues and HICs should focus more on equitable distribution within countries. This study calls for strengthened international partnerships and coordination to address the equitable access to COVID-19 vaccines worldwide, and the WHO should be empowered to take a leading role in guiding more preparedness actions to control the epidemic [31].

According to social media engagement analysis, we found that negative tweets had more followers and higher engagement than positive tweets. This finding might demonstrate that users on Twitter are more interested in communicating and disseminating negative messages such as those expressing misinformation or rumors and lack of vaccine confidence. Previous studies showed that Twitter users sharing misinformation tend to be more connected and clustered [32], and false information travels faster than true information does in social networks [33]. Although scientific experts received considerable attention early on in the COVID-19 pandemic, online attention shifted toward political communities as the pandemic developed [34]. During the evolution of the pandemic, scientific experts lost some of their influence, and it became harder for scientific information to reach a broad audience. In addition, the spread of misinformation or rumors on social media has been found to be significantly associated with vaccine hesitancy [35]. Social media is therefore a double-edged sword that not only can help disseminate public health knowledge directly to the public but also that can, through inappropriate use, be destructive to public health efforts, especially during a public health emergency [36-38]. Therefore, more efforts are needed to build a more proactive public health presence on social media, and health systems should *listen* to tweets from the public to help inform policies related to public health response.

### Limitations

This study captured routine populations who may not be represented in traditional research designs, and social media data can eliminate reporting bias that occurs from speaking with a researcher [20]. However, this study has some limitations. First, there is an inherent bias shared among all social media studies, where users might present themselves differently online or represent a skewed-younger population [39]. Second, we did not extract demographic data because of difficulty to refer to users’ profiles. Third, we used manual coding methods rather than automated annotation, which increased the length of time taken to annotate. Therefore, we only assessed vaccine hesitancy in large metropolises in a short period, and it reduced the generalizability of our findings. In the future, based on our coding data set from this study, we plan to develop a machine learning program to continuously track public attitudes toward COVID-19 vaccination.

## Appendix

Multimedia Appendix 1 Coding framework for COVID-19 vaccine posts on social media.

## Abbreviations

HIC: high-income country
LMIC: lower- and middle-income country
R&D: research and development
WHO: World Health Organization
