# Supplementary material for: Assessing COVID-19 Vaccine Hesitancy, Confidence, and Public Engagement: A Global Social Listening Study
Source: J Med Internet Res. 2021 Jun 11;23(6):e27632. doi: 10.2196/27632 (PMC8202656; doi:10.2196/27632)
Supplement: Multimedia Appendix 1 [file jmir_v23i6e27632_app1.docx]

**Multimedia Appendix 1. Coding framework for COVID-19 vaccine posts on social media**

| **Topics** | **Codes** | **Code definition** |
| --- | --- | --- |
| ***Vaccine hesitancy*** |  |  |
| Attitudes toward COVID-19 vaccination | Accept | Intent to accept, support or be willing to get COVID-19 vaccination. |
|  | Neutral | Neutral, or cannot judge their attitudes. |
|  | Doubt | Concern or doubt about COVID-19 vaccine (e.g. unsafety, ineffectiveness). |
|  | Refuse | Intent to refuse, do not support or be not willing to get COVID-19 vaccination |
| Expectations of COVID-19 vaccine R&D and introduction | Positive | Have confidence towards the research & development and introduction of COVID-19 vaccine; believe or expect that it will be introduced quickly. |
|  | Neutral | Neutral, or cannot judge their attitudes. |
|  | Negative | Doubt or lack of confidence towards the research & development and introduction of COVID-19 vaccine. |
| ***Vaccine confidence*** | | |
| Perceived importance of vaccines | Important | Indicate that COVID-19 vaccine is important, necessary or needed. |
|  | Unimportant | Indicate that COVID-19 vaccine is unimportant, unnecessary or unneeded. |
| Perceived effectiveness of vaccines | Effective | Positive attitudes towards effectiveness (effective, able to produce antibodies or to prevent COVID-19, etc). |
|  | Ineffective | Negative attitudes towards effectiveness (ineffective, unable to produce antibodies, or failed protection). |
| Perceived safety of vaccines | Safe | Positive attitudes towards safety (safe, or no adverse reactions, etc). |
|  | Unsafe | Negative attitudes towards safety (concerns about adverse reactions, or damage to health, etc). |
| Trust in governments | Trust | Trust in government or policy-makers (including all-level government, ministry of health, CDC, etc). |
|  | Distrust | Doubt or distrust in government or policy-makers. |
| Trust in experts | Trust | Trust in experts or professional organization in epidemic prevention field (universities, research institutes, etc). |
|  | Distrust | Distrust in experts or professional organization in epidemic prevention field (universities, research institutes, etc). |
| ***Information around vaccines*** | | |
| Misinformation or rumors |  | Negative information about all vaccines, such as misinformation, rumors, anti-vaccine campaigns, anti-intellectual, anti-science campaigns, vaccine scandals. |
| ***Complacency*** | | |
| Perceived risk of getting COVID-19 | High | COVID-19 is susceptible and severe if suffered, or COVID-19 epidemic is severe and fearful. |
|  | Low | COVID-19 is not susceptible or not severe if suffered (just as severe flu), or COVID-19 epidemic is not severe and there is nothing to fear. |
| ***Vaccine convenience*** | | |
| Vaccine accessibility |  | Mention production or supply capacity of COVID-19 vaccine. |
| Vaccine distribution |  | Mention priority vaccination groups, compulsory or voluntary vaccination. |
| vaccine affordability |  | Mention prices of COVID-19 vaccine, free or not. |
| **Vaccine types** |  |  |
| AstraZeneca |  | Keywords: Britain, AstraZeneca, Oxford university, AZD1222 ChAdOx1 nCoV-19. |
| Moderna |  | Keywords: America, Moderna, mRNA-1273 NCT04470427. |
| Pfizer |  | Keywords: U.S.-German-Chinese cooperation, Pfizer, BioNTech, fosun pharma. |
| Chinese vaccines |  | Vaccines produced by China, including Sinopharm, Sinovac, and undefined Chinese vaccine. |
| **Others** |  | Expressing other views on COVID-19 vaccines and cannot be classified to the above categories. |
| **Irrelevant** |  | Posts without personal opinions (Including News, posts from officials and organizations, quoted without comments, etc), or personal opinions that are irrelevant to the research topic. |
